# Digital transformation of business model innovation

**DOI:** 10.3389/fpsyg.2022.1017750

**Published:** 2022-10-26

**Authors:** Wan-Yi Tsai, Chaun-Jun Su

**Affiliations:** Department of Industrial Engineering and Engineering Management, Yuan Ze University, Taoyuan, Taiwan

**Keywords:** digital transformation knowledge, technology resources, competitive pressure, perceived benefits, perceived risk, innovative atmosphere, digital transformation intention

## Abstract

Because of the rising labor costs and the trade impact after joining the World Trade Organization, the textile industry in Taiwan has encountered many operational and production problems and obstacles. In addition, the lack of knowledge and resources for the digital transformation required to improve business performance has led to poor production and decision-making efficiency, and stagnant R&D for textile product innovation. In order to improve business operations, enhance customer satisfaction and experience, increase efficiency and reduce manual error, this study was conducted with senior executives and company managers in Taiwan’s textile industry as the target population. The knowledge, technological resources, and competitive pressure of digital transformation are the independent variables. The organizational innovation atmosphere is the moderating variable. The study analyzes the impact of digital transformation on the intention of enterprises. The results of the study show that the knowledge of digital transformation, technological resources, and the competitive pressure of the textile industry positively and significantly affect their perceived benefits of digital transformation. Digital transformation knowledge, technological resources, and competitive pressure will negatively and significantly affect the perceived risk of digital transformation. The perceived benefits and risks of digital transformation are related to the intention of digital transformation of enterprises. The organizational innovation atmosphere of the textile industry positively moderates the influence of perceived benefits on digital transformation intention. Finally, based on the research findings, this study provides practical recommendations to senior executives and company managers in the textile industry. It is also recommended to promote the digital transformation of related software and hardware vendors and government agencies, as well as the subsequent related research reference.

## Introduction

From 1970 to 1980, the textile industry was the most important industry and economic pillar in Taiwan. However, the textile industry began to be labeled as the main reason for environmental pollution as the public became more environmentally conscious around 1990 (Chen and Kai-Fang, 2007). In addition, the rising labor cost and the impact of Taiwan’s participation in the World Trade Organization have caused the industry to move out of the country. As a result, the textile industry has been called a sunset industry. To differentiate their products, the textile industry in Taiwan has invested in functional fabric research and development, production, and improvement of production processes and other textile technologies (Lu et al., 2018). To date, there are still more than 4,000 textile factories in Taiwan. More than 140,000 people are employed in the industry (Taiwan Textile Federation, 2021). Although the textile industry can still survive in Taiwan, it still faces overlapping division of labor, inefficient manual production lines, and poor order and equipment management (Huang et al., 2021); most of these are due to inadequate digital management (Hadjitchoneva, 2020). Scholars have therefore proposed digital transformation to help companies improve the aforementioned problems. This includes improving business models and operational processes, improving performance, bringing value to customers, reducing human error, and increasing efficiency (Meena and Parimalarani, 2020).

Recent studies have also mentioned the influences of digital transformation. Bygstad et al. (2017) examined the digital transformation of Scandinavian Airlines in Sweden. The study mentioned the relationship between employees’ digital knowledge and their involvement in digital transformation. In addition, Fenech et al. (2019) investigated the impact of technology resources on the digitalization of companies through interviews with corporate executives in the United Arab Emirates using a qualitative study. The scholars suggest that the technology resources possessed by companies affect the effectiveness of their digitalization policies. However, Lutfi et al. (2022) examined the factors that influence the use of big data by Jordanian SMEs during their digital transformation process. The results show that competitive pressures do not significantly affect the use of big data. Other studies affecting the intention of digital transformation include Lin et al. (2020), which investigated the factors influencing the intention of transformation in Chinese agricultural e-commerce. The results of the study show that the perceived benefits of enterprises positively and significantly affect their e-business transformation intentions. Liébana-Cabanillas et al. (2020) explored the intention to use mobile payment systems among members of the Spanish community. The results showed that the perceived risk negatively influenced the intention to use. Shen et al. (2021) explored the correlation between learning innovation climate and innovation behavior among Chinese nursing students. The results indicated that their learning and innovation climate was positively correlated with their innovation behavior.

The aforementioned study revealed that in addition to competitive pressure, digital knowledge, technological resources, perceived benefits, perceived risks, and organizational innovation atmosphere were all related to or significantly influenced by respondents’ intention to transform digitization. Since the aforementioned study is different from the textile industry, this study still includes competitive pressure in the survey. To summarize the above, this study takes the senior executives and corporate leaders of Taiwan’s textile industry as the target population. The knowledge of digital transformation, the company’s technological resources, and the competitive pressure are the independent variables. The organizational innovation atmosphere is the moderating variable. The impact on the intention of digital transformation is investigated. The objectives of the study are first, to understand the current status of the seven research variables in the textile industry in Taiwan. Second, to confirm the validity of the research questions and assumptions through statistical analysis. Third, to provide practical suggestions to the responsible persons in the textile industry based on the research results. To promote the digital transformation of software and hardware vendors and government agencies, and to provide a reference for subsequent research.

## Literature review and research hypotheses

### Digital transformation knowledge

Liere-Netheler et al. (2018) define digital transformation as the use of new digital technologies. This includes social media, mobile devices, analytics, or embedded devices to achieve significant business improvements. Examples include enhancing the customer experience, streamlining operations, or creating new business models. Goerzig and Bauernhansl (2018) defined digital transformation as the process of change in which firms use computer information technology to enhance competitiveness and develop new value. In summary, this study defines the knowledge of digital transformation as follows. The use of new digital information technologies by textile companies. The knowledge needed to achieve business improvement, enhance competitiveness, and create new business models, such as enhancing customer experience, streamlining operations, and developing new corporate value.

### Perceived benefits

Zheng et al. (2018) defined perceived benefits as the perceived degree to which teaching objectives are accomplished and teaching productivity is enhanced through learning systems. Guidry et al. (2020) performed a quantitative analysis of tweets from the health care sector in 12 countries and defined various methods to effectively reduce personal threats. In summary, this study defines perceived benefits as the perceived extent to which textile companies can reduce corporate threats, achieve corporate goals, and increase product productivity through digital transformation. Moumtzidis et al. (2022) conducted a study on the digital transformation of telecommunication companies through the Internet of things and big data analysis. The results indicate that the data quality knowledge associated with digital transformation positively and significantly affects the perceived benefits of digital transformation systems. Reinartz et al. (2019) examined the threat of digital transformation of online shopping to traditional stores. The study points out that the digital transformation of enterprises includes the personalization of enterprises, the integration of the workplace, and the automation of interactions between departments to improve knowledge and enhance the perceived benefits to customers. In summary, this study concludes that the increase in knowledge of digital transformation in the textile industry will increase its perceived benefits. Therefore, this study proposes the following hypothesis:

H1: The knowledge of digital transformation in the textile industry will positively and significantly affect the perceived benefits of digital transformation.

### Perceived risk

Utami et al. (2018) defined perceived risk as the risk of uncertainty felt when customers cannot predict the consequences of their purchase decisions. Natsir et al. (2021) defined perceived risk as the subjective expectation of loss perceived by an individual on the outcome of behavior. In summary, this study defines perceived risk as the subjective expectation of uncertainty in the textile industry during the digital transformation process. This study defines perceived risk as the subjective expectation of uncertainty due to uncertainty or unpredictability of the transformation outcome. Hemker et al. (2021) interviewed senior managers of companies about their perceptions of digital transformation and analyzed them through qualitative research methods. The results showed that the knowledge level of managers about digital transformation was related to the perceived risk of digital transformation. This study concludes that the perceived risk of digital transformation in the textile industry will decrease as the knowledge of digital transformation increases. Therefore, this study proposes the following hypothesis:

H2: The knowledge of digital transformation in textile industry will negatively and significantly affect its perceived risk of digital transformation.

### Technology resources

Mikalef and Pateli (2016) examine the association between employee technological capabilities and competitive performance. The study defines technology resources as the firm’s technology infrastructure, IT resources related to the firm’s objectives, and the technical skills of employees. Ceric (2016) defines technology resources as technology assets and technology capabilities that prevent firms from threatening and exploiting market opportunities. This study defines technological resources as the technological infrastructure that can be used by the textile industry to avoid threats and exploit market opportunities, information-based resources that are relevant to the textile industry’s objectives, and the IT skills of employees. Kuan and Chau (2001) studied the factors influencing the use of EDI by over 500 small firms in Hong Kong and mentioned that technology resources were associated with perceived benefits. Therefore, this study concludes that the perceived benefits of the textile industry will increase with the increase in technological resources. Therefore, the following hypothesis is proposed:

H3: The technological resources of the textile industry will positively and significantly affect its perceived benefits of digital transformation.

Alotaibi (2014) integrates perceived risk, other factors, and the Technology Acceptance Model. The impact on the use of cloud computing was investigated. The study mentioned that IT resources are related to perceived risk. In this study, it is suggested that the perceived risk in the textile industry will decrease with the increase of technology resources. Therefore, the following hypothesis is proposed:

H4: The technological resources of the textile industry will negatively and significantly affect its perceived risk of digital transformation.

### Competitive pressure

Soewarno et al. (2020) defined competitive pressure as the level of competitive atmosphere in the industry, and Alaskar et al. (2021) defined competitive pressure as the pressure felt by the industry to remain competitive. In this study, competitive pressure is defined as the level of pressure felt by the textile industry from its peers to remain competitive, and the level of competitive atmosphere. Oliveira and Martins (2010) compared the impact of competitive pressures and perceived benefits on the use of e-commerce by firms in the tourism and telecommunications industries in several countries of the European Union. The study mentions that competitive pressure is positively related to perceived benefits. Therefore, this study suggests that the perceived benefits of the textile industry will increase as the competitive pressure increases. Therefore, the following hypotheses are proposed:

H5: Competitive pressure in the textile industry will positively and significantly affect the perceived benefits of digital transformation.

Purwandari et al. (2019) studied the impact of peer competitive pressure, perceived risk, and corporate top management on the use of e-commerce and social networks to enhance business objectives among Indonesian SMEs. The study mentioned that competitive pressure is related to perceived risk. Therefore, this study suggests that the perceived risk of the textile industry decreases as the competitive pressure increases. Therefore, the following hypotheses are proposed:

H6: Competitive pressure in the textile industry will negatively and significantly affect the perceived risk of digital transformation.

### Digital transformation intention

In Ajzen (1991) Theory of Planned Behavior study, intention is defined as the perceived probability of an individual to perform a particular behavior. Ruangkanjanases et al. (2020) defined intention as the indication that an individual performs a behavior and as the antecedent of that behavior. To summarize the above and the previous definitions of digital transformation, this study defines the intention of digital transformation. The perceived probability that a textile company will use new information technology to enhance competitiveness and develop new corporate value behaviors. To increase the intention to shop online, Kim et al. (2004) investigated the factors influencing the antecedents of online search intention. The results of the study confirmed that the perceived benefits of online shopping would positively and significantly affect the intention to shop online. Therefore, this study concluded that the perceived benefits of digital transformation in the textile industry would increase, and the intention of digital transformation would increase accordingly. Therefore, the following hypotheses are proposed:

H7: The perceived benefits of digital transformation in the textile industry will positively and significantly affect their intention to transform digitally.

Tang et al. (2020) analyzed the impact of perceived risk factors on the intention to use financial technology (FinTech, Sabah, Malaysia) among Malaysians. The results of the study indicated that financial risk, legal risk, and operational risk among the perceived risks negatively and significantly affected their intention to use FinTech. Lee and Lyu (2019) investigated the impact of perceived risks and perceived usefulness on IT adoption by using elderly people as the study subjects, and the results indicated that perceived risks of elderly people negatively affect their adoption of self-service technologies. Nguyen and Huynh (2018) analyzed the impact of perceived risk and trust on IT adoption of e-payment and found that perceived risk has a negative impact on IT adoption of e-payment. Therefore, this study concludes that an increase in the perceived risk of digital transformation in the textile industry will lead to a decrease in the intention of digital transformation. Therefore, the following hypotheses are proposed:

H8: The perceived risk of digital transformation in the textile industry will negatively and significantly affect the intention of digital transformation.

### Organizing an innovative atmosphere

Chen et al. (2020) define organizational innovation climate as the extent to which employees perceive that the organization supports and encourages innovative behaviors. He (2013) identified organizational innovation climate as the extent to which employees perceive that the workplace can build new ideas and promote them. In summary, this study defines organizational innovation climate as the extent to which employees in textile companies perceive that their organizations support and encourage new ideas and innovative behaviors. Lei et al. (2021) investigated the effect of authentic leadership on team creativity through an organizational innovation atmosphere in an innovative factory in China. The study investigated the effect of authentic leadership on team creativity through an organizational innovation atmosphere. The results indicated that the organizational innovation climate moderated the effect between authentic leadership and team creativity. Arokiasamy et al. (2021) investigated the impact of management knowledge on corporate innovation technology through organizational innovation culture in Malaysian international firms. The research results show that organizational innovation culture has a moderating effect between management knowledge and corporate innovation technology. The aforementioned studies on team creativity and corporate innovation technology point to the moderating effect of organizational innovation climate and organizational innovation culture. Therefore, this study suggests that there is a moderating effect between perceived benefits, perceived risks, and digital transformation intention in textile organizations. Therefore, the following hypotheses are proposed:

H9: The organizational innovation atmosphere will positively moderate the impact of perceived benefits on the intention of digital transformation.

H10: The organizational innovation atmosphere will positively moderate the impact of perceived risk on digital transformation intentions.

## Materials and methods

**FIGURE 1:**
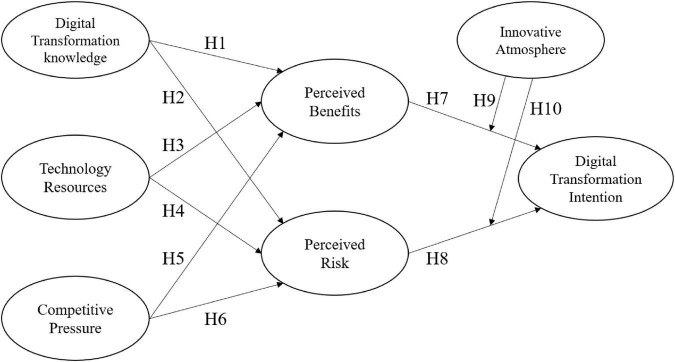
Theoretical model.

This study takes the intention of digital transformation as the main focus. In addition, digital transformation knowledge, technological resources, and competitive pressure are the independent variables. The organizational innovation atmosphere is the moderating variable. The impact on the digital transformation intention of Taiwan’s textile industry is explored. Based on the literature in Chapter 2, the structure of this study is shown in the Figure 1.

### Research subjects and data collection

This study explores the factors influencing the digital transformation intention of Taiwan’s textile industry. The target population was senior executives and corporate managers in the Taiwan textile industry. The survey was conducted using an online questionnaire to collect data. A total of 473 respondents were collected. After removing invalid questionnaires, the total number of valid questionnaires is 450. Based on the proposed formula of sample size requirement by Creative Research Systems (2022), the statistical confidence level of 95% and the confidence interval of 5% were set. The total number of executives and responsible persons in the textile industry in Taiwan, with a sample size of 384, meets the requirement of sample size.

### Measurement scales

The scale investigated the basic personal information of the study subjects, including gender, marriage, education level, and monthly income. A 5-point Likert scale was used to measure their opinions on all study variables. The scale ranged from strongly disagree (1) to strongly agree (5). Expert scholars were invited to review and give their opinions on the questionnaire items after the scale design.

1. Digital Transformation Knowledge Scale: The digital transformation knowledge scale is referred to as the digital transformation scale by Westerman et al. (2014) and the digital business strategy scale by Bharadwaj et al. (2013). Based on the two previous studies, this study modified the digital transformation knowledge scale to include the questions “The company has the necessary knowledge of digital transformation.” and “The company knows using the digital platform.” A total of five measurement items were designed.
2. Technology Resource Scale: The technology resources scale refers to the IT infrastructure resources scale by Mao et al. (2016) and the IT resources scale by Ray et al. (2005). In this study, the technology resources scale was modified to fit the study context to include questions such as “The quality of our company’s digital applications and services can meet the needs of each organization.” and “The company has an adequate technology base for digitization.” A total of five measurement items were designed.
3. Competitive Pressure Scale: The competitive pressure scale is based on the same constructs of Gutierrez et al. (2015) and Habiboglu and Pirtini (2021). In this study, the competitive pressure scale was adapted to the study context with the questions “I am worried that my company will lose its competitive advantage.” and “I am worried that the competitiveness of my peers will overtake my company.” A total of three items were designed.
4. Perceived Benefits Scale: The perceived benefit scale was referred by De Oña et al. (2016) and Kumar and Babu (2017) for the same construct name perceived benefit scale. In this study, the perceived benefits scale was adapted to the study context to include questions such as “I think digital transformation is beneficial to the company.” and “I think digital transformation can improve the quality of the company’s products or services.” A total of five questions were designed.
5. Perceived Risk Scale: The perceived risk scale was referred to Chang (2010) and Shibchurn and Yan (2015) for the same construct name the perceived risk scale. In this study, the perceived risk scale was modified according to the study context to include questions such as “I think that digital transformation will reduce the company’s profitability” and “I am concerned that there will be some unacceptable errors in using the functions of digital transformation.” A total of five measurement items were designed.
6. Organizational Innovation Atmosphere Scale: The Organizational Atmosphere for Innovation Scale is referenced from the Organization support of creativity scale (Saether, 2019) and the Support for Innovation Scale by Siegel and Kaemmerer (1978). To synthesize the two previous studies, this study modified the organizational innovation climate scale according to the study context to include questions such as “Our company’s innovative ideas are respected by our leadership.” and “Our organizations are open to change.” A total of seven measurement items were designed.
7. Digital Transformation Intention Scale: The digital transformation intention scale in this study is based on Piccinini et al. (2015) and the definition of digital transformation intention in Chapter 2 of this study. It also refers to Liébana-Cabanillas et al. (2020) intention to use the technology system scale and Cãpuşneanu et al. (2021) Intention to use Industry 4.0 scale. According to the context, the questions of the previous study scale were “The Company intends to use new digital technologies to achieve significant business improvements.” and “The Company intends to use new digital technologies to enhance customer experience.” A total of four measurement items were designed.

### Data processing and analysis methodology

In this study, the questionnaires were analyzed, and demographic statistics and inferential statistics were examined by using SPSS 23.0 and SmartPLS 3.0 for the valid samples collected. Data analysis methods include the following:

1. Frequency distribution of samples: The distribution and percentages of the sample by gender, marriage, age, education level, and monthly frequency.
2. Outer model analysis: The construct validity of the outer model was examined by the standardized factor loadings, reliability and average variance extracted (AVE). The correlation coefficients between the constructs were compared using the root of the AVE to examine the discriminant validity.
3. Structural model evaluation: The examinations included the goodness of fit test and direct hypotheses between each construct through path analysis. The empirical data were estimated by using Partial Least Squares.

## Data analysis

### Descriptive analysis

The target population of this study was senior executives and responsible persons in the textile industry in Taiwan, with a total of 450 valid samples. In terms of gender, the majority of respondents were male, with 233 respondents accounting for 51.8% of the total number of respondents. In terms of marriage, the majority of respondents were married, with 434 respondents accounting for 96.4% of the total number of respondents. In terms of education level, college and bachelor’s degrees were the most common, with 456 respondents accounting for 34.7% of the total number of respondents. In terms of monthly income, the majority of respondents were above TWD 100,001, with a total of 176 respondents accounting for 39.1%. Table 1 shows that the majority of the monthly income was above 100,001, accounting for 39.1%.

**TABLE 1:** Sample structure.

Category	Label	Frequency	Percentage
Gender	Male	233	51.8
	Female	217	48.2
Marriage	Married	434	96.4
	Unmarried	16	3.6
Education level	High school and below	144	32.0
	College and bachelor’s degree	156	34.7
	Master’s degree and above	150	33.3
Monthly income	30,000 and below	72	16.0
	30,001–50,000	99	22.0
	50,001–100,000	103	22.9
	100,001 and above	176	39.1

### Outer model analysis

The goodness of Fit is an overall indicator of the measured model. When its value is 0.1, it is a weak fit, 0.25 is a medium fit, and 0.36 is a strong fit (Vinzi et al., 2010). In this study, the fit was 0.518, indicating a strong fit.

Assessment of the measurement model includes convergent validity and discriminant validity. As shown in Table 2, the range of factor loadings for all constructs in this study was 0.873–0.947, which were greater than 0.7. The range of Cronbach’s alpha values was 0.782–0.959, which were greater than 0.7. And the range of composite reliability was 0.873–0.970, which were greater than 0.7. Moreover, the range of average variance extracted was 0.610–0.889, all of which were greater than the value of 0.5 suggested by Hair et al. (2010). The results show that all the constructs have sufficient convergent validity.

**TABLE 2:** Reliability and convergent validity.

Construct	Item	Factor loading	Cronbach’s alpha	Composite reliability	Average variance extracted (AVE)
Competitive pressure	CPS1	0.818	0.782	0.873	0.696
	CPS2	0.835			
	CPS3	0.849			
Digital transformation intention	DTI1	0.943	0.959	0.970	0.889
	DTI2	0.947			
	DTI3	0.944			
	DTI4	0.939			
Digital transformation knowledge	DTK1	0.703	0.839	0.886	0.610
	DTK2	0.798			
	DTK3	0.801			
	DTK4	0.835			
	DTK5	0.761			
Perceived benefits	PBT1	0.797	0.851	0.893	0.627
	PBT2	0.748			
	PBT3	0.810			
	PBT4	0.821			
	PBT5	0.780			
Perceived risk	PRK1	0.756	0.863	0.901	0.646
	PRK2	0.838			
	PRK3	0.808			
	PRK4	0.815			
	PRK5	0.797			
Technology resources	TRS1	0.826	0.880	0.912	0.675
	TRS2	0.826			
	TRS3	0.818			
	TRS4	0.816			
	TRS5	0.822			

Fornell and Larcker (1981) present a method for assessing the discriminant validity, which compares the AVE of each construct with the shared variance between constructs. If the AVE for each construct is greater than its shared variance with any other construct, discriminant validity is supported. As shown in Table 3, the diagonal values are greater than the correlation coefficients between the constructs. This indicates that the model has reasonable discriminant validity between the constructs and is suitable for the second step of inner model analysis. In this study, discriminant validity analysis was conducted using the average variance extracted quantity as shown in the Table 3. The diagonal values are larger than the correlation coefficients among the facets, which indicates the discriminant validity among the variables. Also, most of the average variance extracted were larger than the squared correlation coefficients, indicating that the results of this study have discriminant validity.

**TABLE 3:** Discriminant validity.

	CPS	DTI	DTK	PBT	PRK	TRS
CPS	0.834					
DTI	0.248	**0.943**				
DTK	0.585	0.253	**0.781**			
PBT	0.635	0.488	0.559	**0.792**		
PRK	0.536	0.496	0.495	0.664	**0.803**	
TRS	0.585	0.268	0.447	0.548	0.513	**0.821**

CPS, competitive pressure; DTI, digital transformation intention; DTK, digital transformation knowledge; PBT, perceived benefits; PRK, perceived risk; TRS, technology resources. The diagonal value is the square root of AVE. The bold values are the square root of average variance extracted (AVE).

### Inner model analysis

As shown in Table 4, the path coefficient of Digital Transformation Knowledge on Perceived Benefits is 0.249, with a *p*-value = 0.000 < 0.05, so H1 is supported. The path coefficient of Digital Transformation Knowledge on Perceived Risk is 0.233, with a *p*-value = 0.007 < 0.05, so H2 is supported. The path coefficient of Technology Resources on Perceived Benefits is 0.228, with a *p*-value = 0.000 < 0.05, so H3 is supported. The path coefficient of Technology Resources on Perceived Risk is 0.265, with a *p*-value = 0.000 < 0.05, so H4 is supported. The path coefficient of Competitive Pressure on Perceived Benefits is 0.356, with a *p*-value = 0.000 < 0.05, so H5 is supported. Research hypothesis 6: The path coefficient of Competitive Pressure on Perceived Risk is 0.245, with a *p*-value = 0.003 < 0.05, so H6 is supported. The path coefficient of Perceived Benefits on Digital Transformation Intention is 0.285, with a *p*-value = 0.002 < 0.05, so H7 is supported. The path coefficient of Perceived Risk on Digital Transformation Intention is 0.307, with a *p*-value = 0.001 < 0.05, so H8 is supported.

**TABLE 4:** Outer model results.

Hypothesis	Path coefficients	Standard deviation	*T*-values	*P*-values	*Q* ^2^
CPS→PBT	0.356	0.069	5.145	0.000	0.303
DTK→PBT	0.249	0.059	4.214	0.000	
TRS→PBT	0.228	0.085	2.693	0.007	
CPS→PRK	0.245	0.083	2.956	0.003	0.243
DTK→PRK	0.233	0.085	2.722	0.007	
TRS→PRK	0.265	0.092	2.886	0.004	
PBT→DTI	0.285	0.094	3.031	0.002	0.255
PRK→DTI	0.307	0.089	3.464	0.001	

CPS, competitive pressure; DTI, digital transformation intention; DTK, digital transformation knowledge; PBT, perceived benefits; PRK, perceived risk; TRS, technology resources.

**FIGURE 2:**
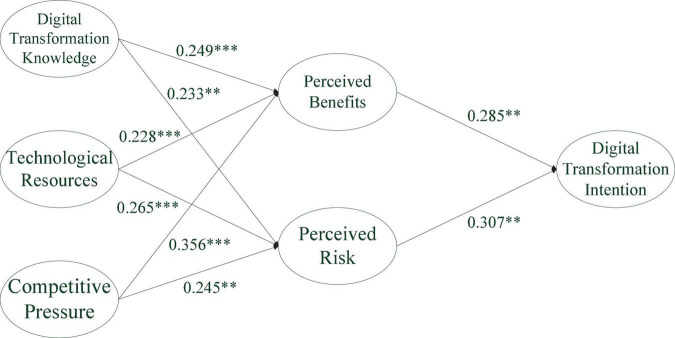
Structural equation modeling (SEM) statistical model diagram. **p < 0.01; ***p < 0.001.

Competitive Pressure, Digital Transformation and Technology Resources explain 49.0% of the Perceived Benefits. The Competitive Pressure, Digital Transformation and Technology Re-sources have the 39.2% exploratory power on the Perceived Risk. Perceived Benefits and Perceived Risk can explain 29.1% of the Digital Transformation Intention. The values of Q-square for Perceived Benefits (*Q*^2^ = 0.303), Perceived Risk (*Q*^2^ = 0.243), and Digital Transformation Intention (*Q*^2^ = 0.255) are greater than zero, indicating that our proposed model has sufficient predictive power (Chin, 1998; Henseler et al., 2009). Figure 2 shows the inner model analysis results.

### Moderating effect

As shown in Table 5, the path coefficient of moderating effect PBT × IAE→DTI is 0.233 (*t*-value = 4.371 > 1.96, *p*-value = 0.000 < 0.05), indicating that H9 is supported. The path coefficient of moderating effect PRK × IAE→DTI is −0.089 (*t*-value = 1.492 < 1.96, *p*-value = 0.136 > 0.05), indicating that H10 is not supported.

**TABLE 5:** Analysis of moderating effect.

Path	Path coefficients	Standard deviation	*T*-values	*P*-values
PBT × IAE→DTI	0.233	0.053	4.371	0.000
PRK × IAE→DTI	−0.089	0.060	1.492	0.136

PBT, perceived benefits; PRK, perceived risk; IAE, innovation atmosphere; DTI, digital transformation intention.

## Conclusion and discussion

### Academic contributions

After empirical analysis, the results of this study validate that knowledge of digital transformation, technological resources, and competitive pressure in the textile industry positively and significantly affect the perceived benefits of digital transformation. These three direct effects were found to be the same or similar to the results of previous studies by Kuan and Chau (2001), Oliveira and Martins (2010), and Moumtzidis et al. (2022), respectively. The results are the same across the different study contexts because the knowledge of digital transformation in the telecom and textile industries has improved and employees are more likely to understand the benefits of digital transformation. Secondly, SMEs and the textile industry have more technological resources and can better understand the benefits of using EDI and digital transformation. In addition, the competitive pressure from the tourism, telecom and textile industries will urge the industry to accelerate the digitalization of their operations. Therefore, they have more experience in IT hardware and software, and can feel more benefits from digital transformation. In addition, the knowledge, technological resources, and competitive pressure of digital transformation in the textile industry negatively and significantly affect their perceived risk of digital transformation. These three direct effects were found to be the same or similar to the results of previous studies by Alotaibi (2014), Purwandari et al. (2019), and Hemker et al. (2021), respectively. Furthermore, the perceived benefits and risks of digital transformation in the textile industry significantly affect the intention of digital transformation positively and negatively, respectively. These two direct effects were found to be the same or similar to the results of previous studies by Kim et al. (2004) and Tang et al. (2020), respectively. In terms of the moderating effect, the organizational innovation atmosphere in the textile industry positively moderates the effect of perceived benefits on the intention to transform digitally. This moderating effect was found to be similar to the results of Lei et al. (2021). The possible reason why H10 is not supported is that there is a part of the textile industry in Taiwan that is resistant to digital transformation. These companies have a lot of senior employees and older executives. These members of the company and the work environment do not have a sense of what an innovative atmosphere is, and therefore do not influence their perceived risk.

### Practical implications

This study combines relevant research with the textile industry context to propose the following recommendations. It is used to strengthen the knowledge, technological resources, and innovation atmosphere of the textile industry for digital transformation, and to enhance the sense of competitive crisis among senior executives and responsible persons. This will increase their competitive pressure and influence their intention of digital transformation. First, regularly hold industrial innovation seminars for employees. This is to enhance the knowledge of digital transformation and to strengthen the digital power of employees. Second, IT department staff should take the initiative to visit each other with information supply chain vendors. Through supply chain vendors, companies can understand the use of digital transformation IT technologies by their peers. By providing the necessary technology resources for the digital transformation of enterprises, the company can reduce time and cost wastage and improve decision-making and production efficiency. Third, the Textile Industry Association (TIA) can help the industry understand customer needs through digital transformation software and hardware vendors. The knowledge, skills, and resources required for innovative product design are identified through the customization process. Through employee training, employees can accumulate confidence in digital transformation, thereby enhancing perceived benefits, and reducing perceived risks. Fourth, companies should eliminate plagiarism and imitation, and provide the necessary resources for innovation. The top management should take the lead in encouraging innovation and forgiving failure. They should not criticize any innovative speech and allow employees to have the freedom of creative ideas to create a good innovative atmosphere.

### Research limitations and future works

This study is subject to some limitations. First, the study was conducted on senior executives and company managers in the textile industry in Taiwan. It did not investigate the attitudes of general employees that might affect the intention of digital transformation. Future research should include employees from different levels. In addition, the attitudes of the study participants toward digital transformation may affect their willingness to fill out the questionnaire. This may affect the validity of the results. Furthermore, to improve the model and further understand the influence of other factors on the intention of digital transformation, other factors that affect the intention of digital transformation in the textile industry can be included in the future. Finally, this study only examines the digital transformation intentions of Taiwan’s textile industry. In the future, the factors influencing digital transformation intentions can be examined from the perspective of different countries and types of enterprises.

## Data availability statement

The raw data supporting the conclusions of this article will be made available by the authors, without undue reservation.

## Author contributions

W-YT and C-JS: conceptualization, data curation, formal analysis, investigation, and writing—original draft. W-YT: writing—review and editing. Both authors have read and agreed to the published version of the manuscript.
